# Supplementary material for: One-Pot Synthesis and Surfactant Removal from MCM-41 Using Microwave Irradiation
Source: Molecules. 2024 Jan 17;29(2):460. doi: 10.3390/molecules29020460 (PMC10820031; doi:10.3390/molecules29020460)
Supplement: Supplementary file 1 [file molecules-29-00460-s001.zip › molecules-2802359-supplementary.pdf]

Supplementary Materials

One-pot synthesis and surfactant removal from MCM-41 by microwave irradiation

Marília R. Oliveira[1], Yasmin T. Barboza[1,2], Thauane S.L. Silva[1], Juan A. Cecilia[3*], Enrique Rodríguez-Castellón[3], Silvia M. Egues[1,2] and Juliana F. De Conto[1,2]

[1]Center for Studies in Colloidal Systems (NUESC), Laboratory of Materials Synthesis and Chromatography, Institute of Technology and Research (ITP), 49032-490, Aracaju-SE, Brazil.

[2]Postgraduate Programme in Process Engineering, Tiradentes University (UNIT), 49032-490, Aracaju-SE, Brazil.

[3]Department of Inorganic Chemistry, Crystallography, and Mineralogy, Faculty of Sciences, University of Malaga, 29071, Málaga, Spain.

*Correspondence: jacecilia@uma.es;

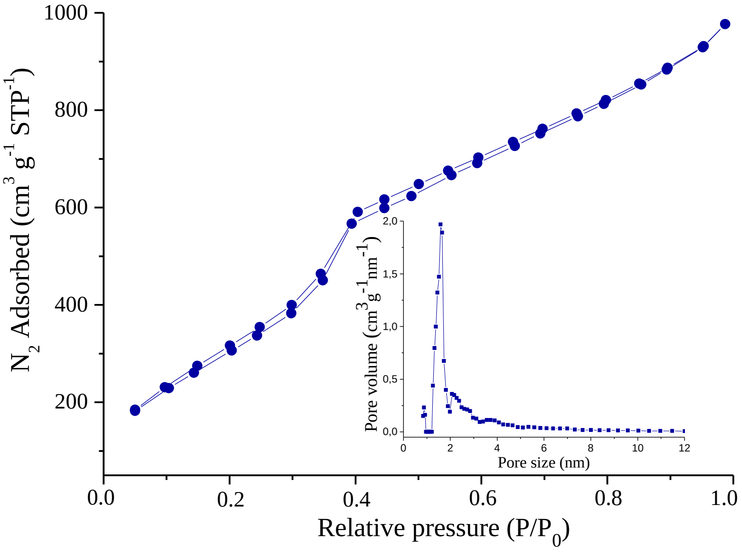

Figure S1. Adsorption/desorption isotherms of silica MCM-41-AC.

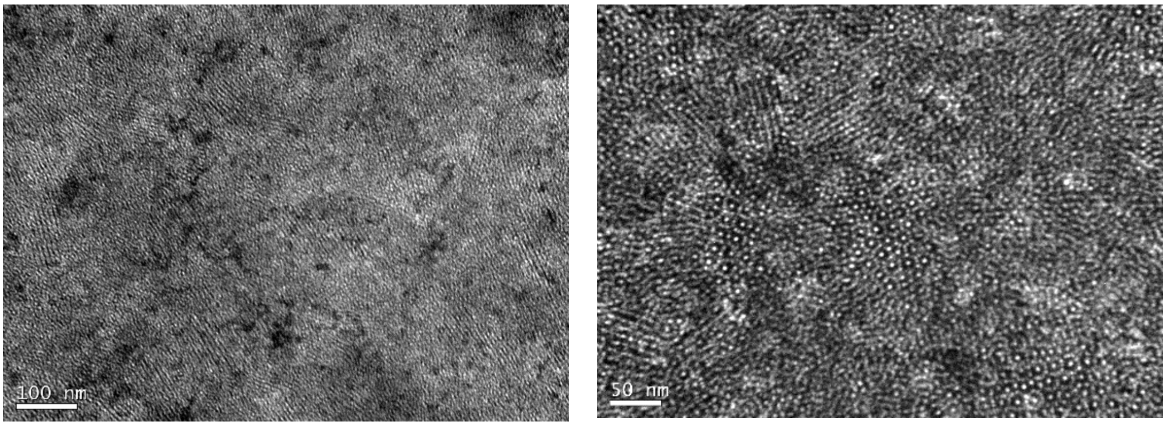

Figure S2. Transmission Electron Microscopy of MCM-41-AC.
